# Ethical Redress of Racial Inequities in AI: Lessons from Decoupling Machine Learning from Optimization in Medical Appointment Scheduling

**DOI:** 10.1007/s13347-022-00590-8

**Published:** 2022-10-20

**Authors:** Robert Shanklin, Michele Samorani, Shannon Harris, Michael A. Santoro

**Affiliations:** 1 Philosophy Department, Santa Clara University, 500 El Camino Real, Santa Clara, CA 950053 USA; 2 Department of Information Systems and Analytics, Santa Clara University, 500 El Camino Real, Santa Clara, CA 950053 USA; 3 School of Business, Virginia Commonwealth University, Snead Hall, 301 W. Main Street, Box 844000, Richmond, VA 23284-4000 USA; 4 Department of Management and Entrepreneurship, Santa Clara University, 500 El Camino Real, Santa Clara, CA 950053 USA

**Keywords:** Ethics, Artificial Intelligence, Machine Learning, Healthcare, Racial Disparities, Bias

## Abstract

An Artificial Intelligence algorithm trained on data that reflect racial biases may yield racially biased outputs, even if the algorithm on its own is unbiased. For example, algorithms used to schedule medical appointments in the USA predict that Black patients are at a higher risk of no-show than non-Black patients, though technically accurate given existing data that prediction results in Black patients being overwhelmingly scheduled in appointment slots that cause longer wait times than non-Black patients. This perpetuates racial inequity, in this case lesser access to medical care. This gives rise to one type of Accuracy-Fairness trade-off: preserve the efficiency offered by using AI to schedule appointments or discard that efficiency in order to avoid perpetuating ethno-racial disparities. Similar trade-offs arise in a range of AI applications including others in medicine, as well as in education, judicial systems, and public security, among others. This article presents a framework for addressing such trade-offs where Machine Learning and Optimization components of the algorithm are decoupled. Applied to medical appointment scheduling, our framework articulates four approaches intervening in different ways on different components of the algorithm. Each yields specific results, in one case preserving accuracy comparable to the current state-of-the-art while eliminating the disparity.

## Introduction 

A range of AI applications give rise to trade-offs between the accuracy of an algorithm’s outputs on the one hand and their fairness or justness on the other hand. Multiple competing definitions of “fairness” are used in articulating and analyzing these trade-offs but in basic terms an algorithm’s outputs may be technically accurate given the data it was trained on yet ethically problematic in one or more ways (Mehrabi et al., 2021). For example, in medical appointment scheduling, algorithms determining which patients should be booked into which appointment slots are typically trained on data that includes who did, and did not, show up late, as well as who did, and did not, show up at all. Owing to a large complex of social, political, and economic reasons, patients of certain ethno-racial identities—for instance Black patients in the USA—have historically been more likely to arrive late, or not at all (Dantas et al., 2018). Current state-of-the-art algorithms then book these patients into less desirable appointment slots, meaning these patients will have to wait longer than other patients, or in extreme cases, perhaps not even see the medical provider (Samorani et al., 2020, 2021). This perpetuates longstanding disparities in access to healthcare.

In this paper, we develop a novel framework for articulating ways of trying to resolve Accuracy-Fairness trade-offs in specific use cases. We apply it to the case of medical appointment scheduling, where the framework helps identify multiple approaches for resolving certain Accuracy-Fairness trade-offs, one of which does so perfectly: particular disparities can be avoided while accuracy remains comparable to the current state-of-the-art. This is achieved by adopting a race-aware approach where the algorithm considers patients’ ethno-racial identities, and then intervening on the Optimization component of the algorithm. Typically, these components have been overlooked as places to intervene as they are often mathematically relatively simple. Applying our framework, we articulate four approaches for intervention and demonstrate how a more complex Optimization component can avoid certain trade-off dilemmas perfectly. Though Accuracy-Fairness trade-offs may not always be avoidable, there is growing reason to think that some, perhaps many, are at least partially avoidable in light of ongoing discussion of how fairness should be defined in AI applications (Corbett-Davies & Goel, 2018; Friedler et al., 2019; Hedden, 2021; Lin et al., 2021; Rastogi, 2021; Rodolfa et al., 2021; Wong, 2020). Our framework, together with the various approaches for intervention it articulates, offers additional promise because of the range of other applications relevantly similar to medical appointment scheduling, where our framework and approaches can in principle be adapted *mutatis mutandis* to those applications.

The paper proceeds as follows. In the next section, we review certain legal aspects of using sensitive data such as ethno-racial identity, as well as arguments for why it is imperative to address ethically problematic algorithmic outputs. In the following section, we present our Decoupling framework and show how it applies to medical appointment scheduling. Then, we show how medical appointment scheduling algorithms perpetuate biases and disparities. Finally, we apply our framework, showing in detail four approaches for intervening to redress ethical disparities in these algorithms, and suggest a range of related applications where our approach could help avoid or reduce Accuracy-Fairness trade-offs.

## Some Ethical and Legal Considerations of Implementing AI Among Systemic Disparities

Accuracy-Fairness trade-offs comprise one AI ethics issue among many, but they arise in a variety of use cases many of which have in common a certain structure that contributes to these trade-offs arising in the first place. That structure is as follows: an algorithm learns from data that reflect disparities including racism/ethnicism, sexism, classicism, ageism, ableism, and homo- and transphobia; the algorithm then proceeds to make predictions or decisions based on those data, and; in doing so, it serves to perpetuate those very disparities (Martin, 2015; Price, 2019; Byrum, 2020; Elyounes, 2020; Kennedy, 2021; Loi & Christen, 2021). The overarching ethical concern is that decisions made or informed by AI in cases with this structure impact peoples’ liberty, access to healthcare, housing, insurance, credit, employment, and transportation, among other social, economic, and political goods (Martin, 2015, 2018, 2019; Mittelstadt et al., 2016).

Medical appointment scheduling is but one instance of use cases with the preceding structure, yet on its own constitutes a serious ethical problem. Historically, low-income patients are disproportionately more likely to show up late or miss medical appointments and being low-income disproportionately correlates in the USA with being Black (Akee et al., 2019; Arrighi, 2001; Bialik, 2018; Creamer, 2020; Hoover & Yaya, 2010; Kaplan-Lewis & Percac-Lima, 2013; Kochhar & Fry, 2014; LeClere & Soobader, 2000; Pollack et al., 2013; Shimotsu et al., 2016). Thus, Black patients in the USA have been disproportionately more likely to arrive late or to miss medical appointments than patients not identified as Black, henceforth “non-Black” following the data we study. Algorithms learning on data reflecting these facts predict that Black patients are less likely to arrive on time or at all. While technically accurate, this gives rise to concerns about fairness; current state-of-the-art algorithms are programmed to maximize overall efficiency for the medical clinics where they are deployed, and as a result produce schedules where Black patients are more likely to wait longer than other patients, or perhaps not even see the medical provider (Samorani et al., 2020, 2021). Since 1619, Black and African American people in the USA (then colonies) have historically had less or no access to the same healthcare as others, or to the same quality healthcare as others, White people in particular (Emling, 2020; Fleming, 2018; Hoberman, 2012; Holloway, 2011). These algorithms have become part of the larger system perpetuating these systemic institutionalized racial disparities among many others, a problem which desperately needs to be addressed (Berard, 2010; Matthew, 2015; Obermeyer et al., 2019). Uses of Big Data and AI algorithms in medicine not only perpetuate but can also exacerbate these disparites, as interactions between patients and providers can lead to self-reinforcing exclusion cycles, wherein Black patients end up having effectively no access to care, or based on experiences with discriminatory practices, may not seek out care in the first place (Bracic et al., 2022). This is particularly problematic from an ethical perspective because it constitutes a feedback loop wherein patients who show up late or not at all then find it increasingly difficult, or even undesirable, to access care going forward (Vamosi et al., 2021).

As will appear, our approaches use data in ways that raise several legal questions. First, explicit use of such data has been regulated in housing, lending, and hiring, among other applications, but has not been regulated in healthcare (Hersch & Shinall 2015). Legal use of specific data to particular ends thus varies from application to application. In 2019, The US FDA issued guidelines suggesting that sensitive data like ethno-racial identity not be used in “critical tasks” like diagnoses, but since appointment scheduling may be a non-critical task, it seems less likely that race-aware approaches like ours would be prohibited (US FDA, 2019; Murray et al., 2020). Second, two of our approaches—one race-aware, one race-unaware—aim to redress inequity by compensating for existing disparate impacts, which arise where a practice employing an algorithm is *prima facie* unbiased yet impacts members of different groups differently in problematic ways. This question has been considered by courts for some time (Ricci v DeStefano, 2009); however, it is not always clear how to apply existing law as it often fails to clearly address discriminatory problems arising from the use of AI (Barocas & Selbst, 2016).

A third legal consideration arises from one of our approaches being race-unaware, which allows for proxy discrimination. This occurs when sensitive or protected data such as ethno-racial identity are taken out of the model, yet other data such as income or zip code correlate strongly enough with ethno-racial identity that the algorithm’s outputs serve to perpetuate disparities. In the USA, while proxy discrimination has been viewed as a form of intentional discrimination rather than of (redressing) disparate impacts, it need not be intentional as when factors are removed from a model explicitly to avoid discrimination against protected categories (Prince & Schwarcz, 2019). Not only is the status of proxy discrimination a nuanced matter, but legalities surrounding proxy discrimination vary among national and international jurisdictions (Martínez-Ramil, 2022). In sum, legal issues arising from applying our framework and associated approaches for intervening on AI vary across use cases and jurisdictions, requiring individual examination based on considerations such as sector, industry, and task.

Many arguments have been given on ethical grounds for intervening on AI in general (e.g., Coeckelbergh, 2020; Floridi et al., 2020; Gabriel, 2022; Hagendorff, 2022; Vallor, 2016), as well as for interventions in specific use cases such as bank loans, student retention, or autonomous cars, among myriad others (e.g., Townson, 2020; Delen, 2010; Lin et. al., 2021). Arguments pertaining to specific cases often focus narrowly on that case, as each case presents specific technical or other details that ethical argument(s) must take into account. This introduces significant complexity to the question of how and whether to ethically intervene. As relates to medical appointment scheduling, many such nuances will emerge in Sects. 3, 4, and 5. Here, we present one argument that may be given for why it is imperative to intervene in our case. The sections below will use our Decoupling framework to articulate how.

A recent and powerful framework for understanding and addressing ethical issues relating to AI is developed in Floridi et al. (2018), and Floridi and Cowls (2022). They survey an array of ethical principles and frameworks, distilling five core principles suited to assessing whether and how AI supports social and environmental goods: Beneficence, Non-maleficence, Autonomy, Justice, and Explicability. Both articles discuss challenges in articulating and applying some of those five core principles, but their ethical framework allows us to argue expediently that the state-of-the-art outcomes we described—booking Black patients into undesirable appointment slots because of ethno-racial identity, thereby perpetuating disparities in access to healthcare—are unethical. At least three of the five core principles are violated. First, these outcomes violate the principle of not doing harm by yielding schedules that not only reduce access to healthcare for Black patients, but moreover do so while perpetuating longstanding systemic institutionalized disparities (Emling, 2020). Second, in doing harm, they also violate the principle of beneficence by failing to promote the well-being of Black patients and by failing to preserve the dignity of all humans regardless of ethno-racial identity. These outcomes also harm businesses and society at large, as missed medical appointments cost billions every year in the USA alone (Sokk & Hall, 2019). Finally, these outcomes violate the principle of justice in virtue of perpetuating unjustifiably unequal treatment, reducing prosperity for Black patients and in doing so ward off solidarity through unequal treatment. Hence, it is over-determined that the current state-of-the-art in AI medical appointment scheduling is ethically highly problematic.

When intervention is ethically imperative, our Decoupling framework may be deployed to clarify and expedite decisions about where and how to intervene on AI algorithms in cases where avoiding or reducing Accuracy-Fairness trade-offs is, or may turn out to be, possible.

## Decoupling AI Components—a Framework for Ethical Interventions

**Fig. 1:**
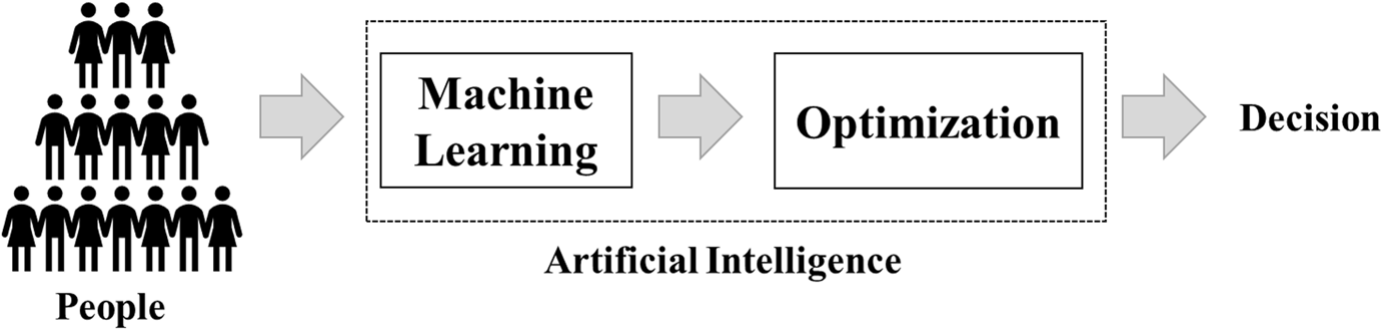
The components of the AI-driven decision-making process

“AI” is an umbrella term for many areas of research and types of algorithm. A current state-of-the-art AI often employed in decision-making processes is composed of two subcomponents (Fig. 1): Machine Learning (ML) and Optimization. The ML component takes data as input and then outputs predictions, such as the probability that a patient will show up on time for their appointment. The Optimization component uses those predictions as input and then yields outputs that optimize for certain factors, in this case schedules that minimize overall patient waiting time, as well as provider down-time and overtime. Typically, Optimization components are mathematically rather simple, as in this case, or even simpler as in some lending cases where an Optimization component merely selects all loan applicants that the ML component predicts as being above a certain threshold of probability for repaying loans.

Though Optimization components are familiar, intervening on them has not been a standard approach for addressing biases and inequities. Our framework explicitly articulates distinct stages for independent interventions aimed to integrate ethics with AI: in our case, at the ML stage and at the Optimization stage. We will show in this and the next section how each of these interventions could be used to compensate for disparities or discrimination that may occur if ML predictions are substantially different for different groups of people, or when an Optimization exacerbates disparities of the ML predictions.

Applying our framework may involve choosing among the approaches it articulates for ethically intervening on AI. If the ML component produces high-quality and accurate predictions, then it is generally desirable to preserve that accuracy. In that case, the optimal way to redress disparities is at the optimization phase. Conversely, when the ML’s predictions are less accurate, then the optimal place to intervene is at the ML component. As relative accuracy is a sliding scale, there may be cases where it is less clear which approach is optimal, so either or both interventions may be considered, including altering the training data for the ML (Allen et al., 2020).

Intervening at the ML component in our case involves modifying the prediction model so that the predictions it generates become less determined by factors correlated with ethno-racial background. One such intervention consists of removing predictors that correlate with race, such as zip code, income, and employment status. This intervention clearly has the potential to reduce disparity. However, because those factors do indeed correlate with a patient’s ability to arrive on time, removing them from the statistical model typically results in a lower prediction performance of the ML. Those outputs would consequently result in sub-optimal decisions, obviating practical benefits of adopting the algorithm in the first place, yielding an accuracy trade-off.

The second intervention point is the Optimization component. This type of intervention consists of changing one of its subcomponents to try to reduce disparity, in our case, the objective function, that involves altering what the Optimization component aims to optimize for. On this intervention, the ML component may remain unchanged, so that predictions may remain as accurate as possible by being based on as much data as possible. We will demonstrate how to alter the objective function so that the Optimization component does redress disparities by compensating for any bias in the ML’s predictions. This type of intervention can be done either “explicitly” or “implicitly.”

Minimizing disparity explicitly consists of employing ethno-racial information in the Optimization model. In our case, this type of intervention could consist of ensuring that Black and non-Black patients have the same probability of longer wait times; this objective is also known as “statistical parity” (Verma & Rubin, 2018). Another way to minimize disparity explicitly consists of adding constraints on the Optimization component that forbid decisions that disproportionately, or excessively disproportionately, allocate undesirable appointment slots to members of one group among all patients seeking appointments. For example, if 30% of the patients are Black and 70% non-Black, then the added constraints can consider only solutions where at least 20% of the desirable slots are given to Black patients and, for parity, at least 60% to non-Black patients.

Minimizing disparity implicitly consists of changing the Optimization model without using ethno-racial information, in the hopes of reducing injustice as a secondary effect. In our case, this type of intervention can be done by including an extra constraint that only allows decisions where, for instance, at least 20% of desirable appointment slots are allocated to patients whose risk of no-show is in the highest third of patients seeking appointments. This may be justified on the grounds that this turns out to unintentionally correlate with ethno-racial background. By forcing some relatively high-risk patients to be approved, this intervention is likely to reduce racial disparity to the degree that risk of lateness or no-show is indirectly correlated with ethno-racial identity.

**Fig. 2:**
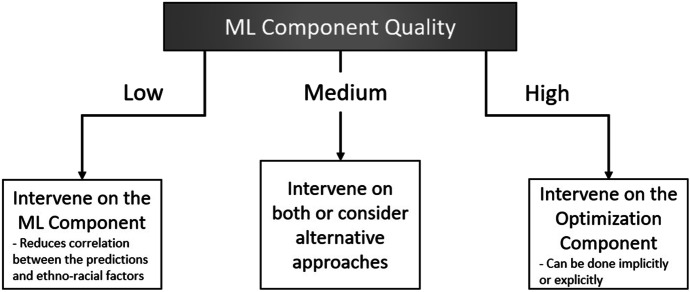
A framework for integrating ethics with AI

Our framework for assessing when and how to intervene in certain types of AI algorithms decouples interventions in the ML component from those in the Optimization component as illustrated in Fig. 2. Step one is to determine the quality of outputs from each component of the AI. This helps identify which interventions will most likely avoid accuracy losses. Decoupling components of the algorithm—in our case the ML component from the Optimization component—also allow us to more efficiently and accurately represent options available for avoiding or minimizing unfairness, such as perpetuating systemic institutionalized racism. This decoupling has been largely neglected by the extant AI literature, we think most probably because typically the Optimization component is mathematically trivial. In many applications, it simply selects the top $x$-number of predictions output by the ML. For example, in AI-driven hiring decisions, the ML component predicts the likelihood that each candidate turns out to be a “good hire” (based, e.g., on past hiring and performance data), and the Optimization component simply selects the top candidate(s), usually in ranked-order.

## How and Why Algorithms Yield Racially Biased Outcomes

In this section, we discuss state-of-the-art appointment scheduling systems in more detail, explaining why systems deployed in this use case are likely to result in racial disparity. We also discuss possible technical interventions to redress disparities facing Black versus non-Black patients, specifically.

**Fig. 3:**
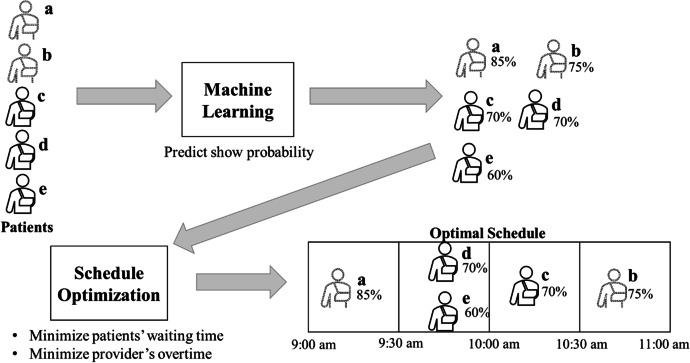
The predictive overbooking framework

Patient no-shows are one of the main challenges faced by medical clinics when scheduling appointments. No-shows are disruptive to the clinic and result in inefficiency, including provider underutilization. One of the main strategies to counteract these ill effects is to predictively overbook appointments, which means assigning the same appointment time to more than one patient, with the expectation that some patients will not show up (Zacharias & Pinedo, 2014) (this may be familiar from similar techniques used to increase efficiency in airline bookings). Because the probability of showing up varies significantly from patient to patient, state-of-the-art appointment scheduling systems implement a framework known as “predictive overbooking,” which employs ML to predict each patient’s individual probability of showing up. The predictive overbooking framework is depicted in Fig. 3. The patients depicted in dotted lines are in the high show probability group (e.g., non-Black patients); the patients depicted in solid lines are in the low show probability group (e.g., Black patients).

In Fig. 3, five patients are scheduled into four 30-min appointment slots of a clinic session that runs from 9:00 to 11:00 am. In this example, patients *c*, *d*, and *e* are Black, while patients *a* and *b* are non-Black. The ML predicts each patient’s individual show probability, which for the reasons discussed in Sect. 2 correlate with race. Then, the Schedule Optimization component uses these predicted probabilities as input to assign the patients to appointment slots. The objective is to find a schedule that minimizes schedule cost, defined as minimizing overall patient wait time, together with provider down-time and provider overtime. The weighted sum of these three components is defined as “schedule cost.” In the rest of the paper, we refer to “schedule quality” to denote the opposite of the schedule cost: the lower the schedule cost, the higher the schedule quality.

It has been shown that efficiency is maximized by placing the patients with the lowest predicted show probabilities into either an overbooked slot (patients *d* and *e* in Fig. 3) or in the slot right after an overbooked slot (patient *c*) (Zacharias & Pinedo, 2014). These appointment slots are undesirable because they are associated with longer waiting times. For example, if both patients *d* and *e* show up, one of them will have to wait for 30 min, and this delay will also affect patient *c* if s/he shows up. In contrast, patient *a*, who is non-Black, does not have to wait in any circumstance; patient *b*, also non-Black, will have to wait only in the unlikely event where patients *c*, *d*, and *e* all show up. Because of the correlation between race and no-shows, patients scheduled in undesirable appointment slots (*c*, *d*, and *e* in Fig. 3) predominantly belong to members of the marginalized racial group.

## Decoupling to Redress Disparities

In this section, we deploy our Decoupling framework from Sect. 3 to address Accuracy-Fairness trade-offs exemplified by the scenario from Sect. 4: whether and how we can reduce unethical disparities while maintaining the practical benefits of using AI in medical appointment scheduling. In doing so, we will examine four ways of integrating ethics and AI, and we will demonstrate how one of these four avoid trade-offs by satisfying ethical considerations at the same time as practical ones.

**Fig. 4:**
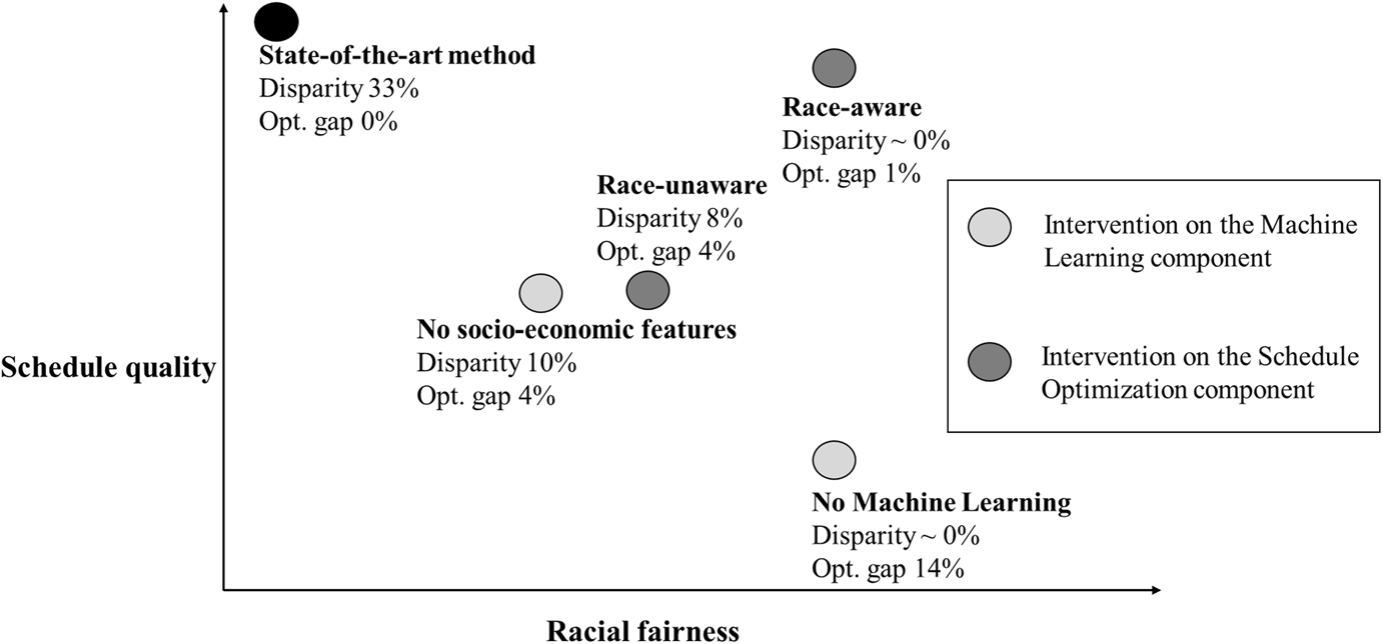
Performance of the methodologies developed by Samorani et al. (2021)

Figure 4 represents four approaches for intervening to integrate ethics and AI, as well as their respective performance as regards increasing efficiency while reducing disparity. The performance of each method is graphed along two dimensions: racial fairness, measured as a percent difference between average wait times of Black versus non-Black patients, and schedule quality, again measured as the opposite of schedule cost. Maximum schedule quality is achieved by the current state-of-the-art method because it aims only to maximize the schedule quality without concern for fairness. Next to each strategy in the chart, we quantify the schedule quality obtained by reporting the optimality gap, or loss of efficiency, relative to the state-of-the-art method. The lower the optimality gap, the higher the schedule quality.

According to our framework from Sect. 3, there are two distinct ways to reduce wait time disparity in appointment scheduling: intervening on the ML component or the Schedule Optimization component. The former aims at reducing the correlation between predicted show probabilities and ethno-racial identity, while the latter aims at reducing disparity by explicitly or implicitly compensating for it in the objective function within the Schedule Optimization component. In our scenario, we consider two different interventions for each intervention type, as illustrated in Fig. 4:

Intervention on the Machine Learning Component (light gray dots):

1. No Machine Learning
2. ML but with no socio-economic features

Intervention on the Schedule Optimization Component (dark gray dots):

3. Race-aware approach
4. Race-unaware approach

The following sub-sections discuss each intervention in more detail.

### No Machine Learning

The simplest way to try to remove disparity is to remove the ML component altogether from the predictive overbooking framework. Under this approach, individual predictions are simply not made, so that all patients have the same show probability, equal to the overall population show rate. On one hand, removing the ML component results in zero disparity as all patients are treated the same by the Schedule Optimization component. On the other hand, though, the quality of the resulting schedule is 14% lower than obtained by the state-of-the-art method because this method ignores that patients may have different no-show behavior.

### No Socio-economic Features

A less extreme way to try to reduce the racial disparity involves keeping the ML component, but without using any socio-economic or other sensitive features when making predictions. The goal is to leverage some of the available information that may help predict show probabilities, such as the appointment’s day of the week or the patient’s past no-shows, while excluding socio-economic features that are well-known to correlate with race, such as the patient’s zip code, marital status, and employment status. This is an approach to correct for bias contained in the data—Big Data bias—by eliminating socio-economic factors. However, despite the exclusion of these features, this strategy still yields some disparity—10% in Samorani et al. (2021)’s experiments—as features summarizing the patient’s no-show history still correlate with ethno-racial identity. The schedule quality is, on average, 4% lower than the state-of-the-art method, because the show predictions are less accurate if some features are excluded. Thus, in the avoidance of Big Data bias, algorithmic bias emerges as the algorithm extracts patterns of correlation between non-socio-economic factors and socio-economic factors (Hajian et al., 2016; Richardson et al., 2019).

### Race-Aware Approach

This approach modifies the Optimization component to minimize disparity *explicitly* by changing the objective function (OF) to use data on ethno-racial identity to minimize ethno-racial disparity. The ML component is the same as in the state-of-the-art method, so predicted show probabilities correlate with ethno-racial identity; Black patients are predicted to have a lower show probability.

Racial disparity in the form of longer wait times is then reduced by adopting a race-aware OF instead of the traditional OF in the Schedule Optimization component. While the traditional OF minimizes the wait time of *all patients*, the race-aware objective function minimizes the wait time of the patients belonging to the racial group expected to wait longer. As illustrated in Fig. 4, the results by Samorani et al. (2021) show that a race-aware OF results in a nonsignificant racial disparity and in a schedule quality that is only 1% worse than that obtained by the state-of-the-art OF.

### Race-Unaware Approach

Though our race-aware approach avoids Accuracy-Fairness trade-offs effectively by simultaneously maximizing efficiency while effectively eliminating racial disparity, some practitioners may be reluctant to adopt a race-aware, or “non-colorblind” approach. An alternative is the race-unaware approach, which does not take ethno-racial identity into account explicitly, but still aims at helping the least advantaged group. Mathematically, the race-unaware objective function minimizes the wait time of the individual patients expected to wait longest.

As illustrated in Fig. 4, the results by Samorani et al. (2021) show that the race-unaware OF approach largely avoids Accuracy-Fairness trade-offs, though not quite as effectively as the race-aware approach: disparity is present though at a lower-than-state-of-the-art 8%, and there is a 4% loss in efficiency. Importantly, both our race-aware approach and our race-unaware approach are examples of ethically effective AI interventions that reduce (race-unaware) or even eliminate (race-aware) certain Accuracy-Fairness trade-offs.

### Further Possible Applications

To illustrate how our framework may be applied to other cases, consider a simplified access-to-credit example. A set of people apply for loans at a bank, and the bank uses an algorithm that selects the subset of customers for whom to approve the loan. The ML component predicts the risk of default for each customer: the probability that they fail to repay the loan. These predictions are then inputs for the Optimization component, which on a standard approach finds the subset of loans to approve that will maximize expected profit for the bank. The probability of default for each customer is clearly an important input to the overall system, and it is very likely that probability of default correlates with the customer’s socio-economic background, which is in turn correlated in many parts of the world with ethno-racial status. It should be clear that if the goal of the Optimization component is simply to maximize profit, then the decisions made will inevitably penalize members of the ethno-racial groups that the ML associates with higher risks of default, as it is obviously optimal for the bank to lend money only to the customers with the lowest risks of default. Applying our framework articulates choices analogous to those above for medical appointment scheduling, and then decisions can be made about how best to intervene given the specific technical and legal realities relating to decisions about approving credit.

Three of our approaches apply to this access-to-credit example as follows. One race-unaware intervention could consist of removing predictors that are correlated with race, such as zip code and employment status. This intervention clearly has the potential to reduce disparity. However, as zip code and employment status correlate with many customers’ ability to repay debt, removing those data from the statistical model should result in a lower prediction performance of the ML. We predict that intervening on the Optimization component’s objective function would yield more attractive results. One race-aware intervention on the Optimization component could be the imposition of “race-based” quotas: for example, if 30% of the applicants are Black and 70% non-Black, then the new constraints could consider only solutions where at least 20% of the approved applications are from Black applicants and at least 60% are from non-Black applicants. A third, race-unaware, approach could impose a similar constraint using “risk of default” as criterion, which is different from race but correlated to it: for example, approving at least a certain number of applications from applicants whose risk of default is in the highest third of the customers applying for loans.

Our four approaches to the medical appointment scheduling, and three approaches to the simplified access-to-credit example, are visualized in Table 1. It illustrates decoupling, which distinguishes our framework from others: the independent treatment of components and subcomponents of AI algorithms—in this case, the ML component and the Optimization component’s objective function. A general framework for ethical interventions on AI has been developed by Lin et al. (2021), but it is unclear how that framework distinguishes independent interventions on components and subcomponents within AI algorithms, as it articulates interventions on algorithms in terms of input- and output-based. The two frameworks can, in principle, be synthesized to articulate additional approaches for intervention, beyond an algorithm’s input and output stages.

**Table 1:** Decoupling framework for intervention on AI

Application	Intervention	AI Component	Race-aware?
Access to credit	Race-based quotas	Optimization	Yes
Access to credit	Risk-of-default-based quotas	Optimization	No
Access to credit	Remove socio-economic features when predicting ability to repay the loan	Machine Learning	No
Appointment scheduling	No socio-economic features	Machine Learning	No
Appointment scheduling	Attempt to equalize waiting times of patient at highest risk of no-show and at lowest risk of no-show	Optimization	No
Appointment scheduling	Attempt to equalize waiting times of Black and non-Black patients	Optimization	Yes

By adapting our framework to accommodate varying technical and legal realities, it can be applied to an assortment of cases structurally similar to medical appointment scheduling, wherein Accuracy-Fairness trade-offs emerge yet there is the possibility, or at least hope, for minimizing or avoiding them. For example, in medicine alone, applications include image analysis in radiology and ophthalmology, identification of malignant lesions in dermatology, and identification of cancers in pathology (Jiang et al., 2017; Mittelstadt, 2019; Yu et al., 2018). Algorithms have also been used in emergency medicine, for instance aiming to reduce patient wait times (Tang et al., 2021). Applications in education include student retention, and in the public sector, include algorithms to manage and reduce the spread of COVID-19, as well as to support judicial decisions on bail and sentencing (Delen, 2010; Henman, 2020; Sourdin, 2018; Vaishya et al., 2020). Applications in business, in addition to access to credit, include algorithms to reduce insurance fraud, as well as to determine insurance premiums and coverages (Cannings, 2021; Jiang et al., 2019; Townson, 2020).

Critics may worry that implementing race-aware approaches serves to re-introduce racism into the algorithm, where many hoped that using of AI would avoid such biases. Such squeamishness may be understandable given public discourse around racism and other disparities, but attempting to be “colorblind” is critically misguided. Experts, including Black activists and scholars, agree: trying to be colorblind helps maintain systemic institutionalized racism in the status quo by attempting to ignore signs of racism (Burke, 2018; Yi et al., 2022). It is a psychological fiction to think that human beings can just set aside the pressures and realities of systemic institutionalized racism in general, including in medicine (Hoberman, 2012; Alexander 2010; Braddock 2020; Eberhardt, 2020). Moreover, for many decades, scholars and others too numerous to represent here have argued that it is ethically insufficient to try to be “colorblind” or in some other way non-racist—rather, to be on the right side of racism and other disparities, one must be actively anti-racist (e.g., Baldwin, 1962; King, 1963; Bell, 1992; Applebaum, 1997; Mills, 2007; Moule, 2009; Berard, 2010; Gines (Belle), 2010; Dotson, 2015; Ross, 2016; Haslanger, 2017; Kendi, 2019; Boykin et al., 2020; Coe, 2020; Igbokwe, 2021). This involves working to resist, subvert, and change racist systems and institutions, including AI applications in medicine among other fields. This should underline the ethical imperative to intervene wherever possible. Otherwise, those structures perpetuate racism and other disparities both in general and in AI applications (Bayer, 2022; Benjamin, 2019; Floridi et al., 2020; Wellner & Rothman, 2020). Hence, while we do not take an explicit stance on which particular approach of intervening on the AI is ultimately “best” for any particular use case, we emphasize the importance of overcoming squeamishness about race-aware approaches in AI.

## Conclusion

Accuracy-Fairness trade-offs emerge across a range of AI applications. On the one hand, AI can use vast amounts of data as input, and as a result of superior pattern recognition, offer many practical benefits, such as increased accuracy and efficiency over non-AI approaches. In our study, it reduces both overall patient wait time as well as provider down-time and overtime when used to schedule medical appointments. On the other hand, existing data sets reflect biases such as ethno-racism, homo- and transphobia, sexism, classism, ageism, ableism, and many others. Such biases, among other factors, have resulted in certain groups being historically less likely to arrive on time or at all to medical appointments. As the AI is trained on those data sets, it becomes complicit in perpetuating systemic institutionalized disparities, in our case by assigning disproportionately longer wait times, in particular to Black patients than non-Black patients. Skeptics, including in the public realm, sometimes assert that the AI is not inherently biased, or that problems with the data are not strictly speaking “AI problems,” or both. True or not, both positions miss the point and misunderstand the issues. The issues our argument engages concern neither blaming algorithms nor branding them “racist” per se, but rather that it is ethically imperative to intervene on these algorithms, and that our framework articulates often-unconsidered approaches for effective ethical interventions.

Our framework decouples components and subcomponents of AI algorithms used in a range of applications: the ML component that makes predictions and the Optimization component that chooses outcomes. This allows multiple ways to intervene on algorithms in ways that can reduce or avoid Accuracy-Fairness trade-offs. Ethical and legal considerations such as those raised in Sect. 2 indicate when and why we ought to intervene to address disparities by revealing the nature and severity of disparities involved in any particular case in which AI is used or proposed for use. In the specific case of medical appointment scheduling, intervening on the Optimization component by altering its objective function proved the most effective way to reduce disparity while preserving efficiency in the form of schedule quality. The goal of the intervention was to minimize patient wait time, thereby reducing and effectively eliminating certain ethno-racial disparities. In a variety of AI applications sharing the structure that contributes to Accuracy-Fairness trade-offs, similar interventions—adjusted for the specifics of each application—can be developed. In whatever case under consideration, one can assess the relevant legal and ethical considerations as in Sect. 2, then use the Decoupling framework from Sect. 3 to assess how best to intervene on the algorithm in question to reduce the disparities relevant to each application. In our case, both the race-aware and race-unaware approaches to intervention outperformed the current state-of-the-art method in terms of reducing disparity, but the race-aware approach best avoided trade-offs, as it yielded overall efficiency to within ~ 1% of the current state-of-the-art.

## Data Availability

None of the graphs/visuals/tables used in this paper appears elsewhere. This paper discusses data and results from two earlier papers; one on which one of the authors worked, the other on which three of the authors worked. As best as the Corresponding Author can see, none of those data appears in the same form in this paper. The more earlier paper on which we draw more heavily is Open Access: Samorani, M., Harris, S., Blount, L.G., Lu, H. and Santoro, M.A., 2021. Overbooked and overlooked: machine learning and racial bias in medical appointment scheduling. *Manufacturing and Service Operations Management Articles in Advance.* https://pubsonline.informs.org/doi/epdf/10.1287/msom.2021.0999
